# Innovative Peptide Therapeutics in the Pipeline: Transforming Cancer Detection and Treatment

**DOI:** 10.3390/ijms26146815

**Published:** 2025-07-16

**Authors:** Yanyamba Nsereko, Amy Armstrong, Fleur Coburn, Othman Al Musaimi

**Affiliations:** 1 School of Pharmacy, Newcastle University, Newcastle upon Tyne NE1 7RU, UK; 2 Department of Chemical Engineering, Imperial College London, London SW7 2AZ, UK; 3 Orthogonal Peptides Limited, London SW7 2AZ, UK

**Keywords:** cancer, peptides, targeted therapy, theranostic, diagnostic

## Abstract

Cancer remains a leading global health burden, profoundly affecting patient survival and quality of life. Current treatments—including chemotherapy, radiotherapy, immunotherapy, and surgery—are often limited by toxicity or insufficient specificity. Conventional chemotherapy, for instance, indiscriminately attacks rapidly dividing cells, causing severe side effects. In contrast, peptide-based therapeutics offer a paradigm shift, combining high tumour-targeting precision with minimal off-target effects. Their low immunogenicity, multi-pathway modulation capabilities, and adaptability for diagnostics and therapy make them ideal candidates for advancing oncology care. Innovative peptide platforms now enable three transformative applications: (1) precision molecular diagnostics (e.g., ^18^F-PSMA-1007 for prostate cancer detection), (2) targeted therapies (e.g., BT5528 and SAR408701 targeting tumour-specific antigens), and (3) theranostic systems (e.g., RAYZ-8009 and ^177^Lu-FAP-2286 integrating imaging and radiotherapy). Despite their promise, peptides face challenges like metabolic instability and short half-lives. Recent advances in structural engineering (e.g., cyclization and D-amino acid incorporation) and delivery systems (e.g., nanoparticles and PEGylation) have significantly enhanced their clinical potential. This review highlights peptide-based agents in development, showcasing their ability to improve early cancer detection, reduce metastasis, and enhance therapeutic efficacy with fewer adverse effects. Examples like CLP002 underscore their role in personalised medicine. By overcoming current limitations, peptide drugs are poised to redefine cancer management, offering safer, more effective alternatives to conventional therapies. Their integration into clinical practice could mark a critical milestone in achieving precision oncology.

## 1. Introduction

Cancer is a leading global cause of death, marked by uncontrolled cell growth and proliferation [1]. This pathological progression can result in metastatic dissemination to distant organs, significantly complicating therapeutic interventions [1]. Current treatment modalities, including radiotherapy, chemotherapy [2,3], immunotherapy [4], and molecular-targeted therapy, aim to suppress tumour progression [1,5,6]. While surgical resection is effective for localised solid tumours, incomplete removal may necessitate repeated procedures, potentially increasing patient morbidity and mortality risk [7]. A critical limitation of conventional therapies, particularly chemotherapy, is their poor cellular selectivity [5]. These treatments indiscriminately target rapidly dividing cells—both malignant and healthy populations in tissues such as hematopoietic systems, gastrointestinal epithelium, and hair follicles—resulting in substantial systemic toxicity and adverse effects [5].

Peptides play a pivotal role in targeted cancer therapy by specifically binding to overexpressed receptors or molecules within the tumour microenvironment (TME) [8,9]. They function either as therapeutic agents themselves or as targeted delivery vehicles for cytotoxic payloads that induce DNA damage or inhibit cell division [8,9]. Compared to conventional chemotherapy, peptide-based therapies demonstrate significantly fewer side effects due to their high target specificity [8,9,10]. This enhanced precision not only improves treatment efficacy but also substantially enhances patient quality of life by minimising off-target toxicity.

**Figure 1:**
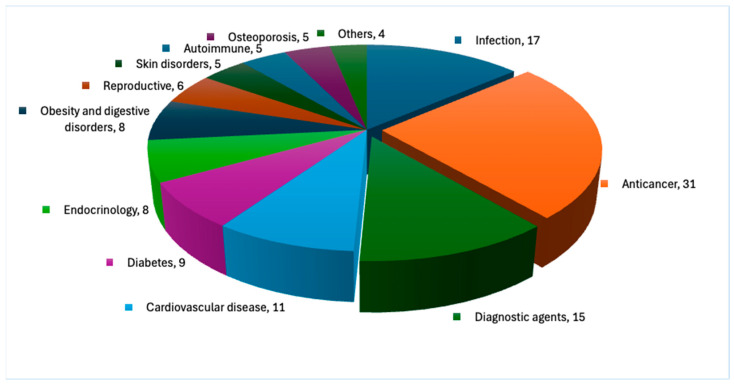
Total number of FDA-approved peptide-based therapeutic applications between 1989 and 2025.

Peptides are biologically active polymers composed of up to 50 amino acids, with molecular weights ranging from 500 to 5000 Da [5]. Beyond their structural properties, peptides play a significant role in biomedical applications, including diagnostics [11], immunomodulation [12], and pharmaceutical development [13,14]. In oncology, peptides serve as versatile agents with theranostic, diagnostic, and therapeutic potential, enabling precise tumour targeting and treatment [9]. Their utility extends beyond cancer therapy, with applications in diverse medical fields such as urology, respiratory medicine, pain management, metabolic and cardiovascular disorders, and antimicrobial therapy (Figure 1) [15,16,17].

To minimise systemic toxicity and enhance cellular specificity, increasing research efforts are being directed toward boosting peptide-based targeted therapies, particularly antibody–drug conjugates (ADCs) and peptide–drug conjugates (PDCs) [18,19]. These advanced therapeutic modalities aim to improve treatment efficacy while reducing off-target effects, ultimately leading to better patient outcomes and quality of life [20,21,22].

Peptide-based therapeutics have demonstrated clinical efficacy across a spectrum of malignancies, including hepatocellular carcinoma, advanced metastatic cancers, hormone-responsive cancers (such as breast and prostate cancer), non-small cell lung cancer (NSCLC), and various solid tumours (Table 1) [9]. These targeted therapies offer promising alternatives to conventional treatments, particularly for tumours with specific molecular markers.

This review provides a systematic analysis of (i) the classification of therapeutic peptides, (ii) approved peptide-based drugs, and (iii) investigational candidates under clinical development. For each category, we evaluate molecular mechanisms of action, structural features, biological targets, routes of administration, therapeutic monitoring criteria, and adverse effect profiles. A dedicated focus on structure-activity relationships (SAR) bridges molecular design with clinical utility, underscoring their relevance across diverse therapeutic areas. Illustrative examples from Table 1 are discussed to highlight mechanistic insights and translational challenges in peptide drug delivery.

## 2. Peptide Classes

Since 1989, several distinct classes of therapeutic peptides have been developed for oncological applications. These include gonadotropin-releasing hormone (GnRH) analogues, somatostatin analogues (SSAs), PSMA-targeting peptides, ADCs, PDCs, and peptide receptor radionuclide therapy (PRRT), among others [9]. Moreover, certain peptide therapeutics like pegulicianine (Lumisight) demonstrate unique mechanisms of action that do not conform to these established classifications, representing the novel categories of peptide-based agents [7,64].

Peptides combat cancer through multiple mechanisms, such as acting as receptor agonists or antagonists to alter signalling pathways [65], inhibiting enzymes essential for tumour survival, and conjugating with radionuclides for diagnostic imaging or targeted radiotherapy [66,67]. Their versatility—particularly in theranostic applications where a single peptide can both diagnose (via radiolabelled imaging) [66,67] and treat (through targeted radiation or drug delivery)—makes them highly valuable in precision oncology [9,68]. For example, SSAs not only activate tumour-suppressive pathways but also deliver radioactive isotopes for detecting and treating neuroendocrine tumours. Similarly, RGD-based peptides selectively target tumour vasculature for dual imaging and therapeutic effects [69,70].

Thanks to their multifunctionality, high specificity, and low toxicity, peptides have become essential tools in modern cancer therapy, especially for overcoming drug resistance and enabling personalised treatments via targeted delivery systems [9,71]. Ongoing advancements in peptide conjugates that combine diagnostic and therapeutic functions are expected to expand their clinical applications in oncology [20,68,72]. Furthermore, peptides are increasingly recognised as potent anticancer agents due to their precision in disrupting critical tumour-driving pathways, low toxicity, and ability to target protein–protein interactions (PPIs) [73,74], enzymes [75], and receptor signalling [65]—key mechanisms that sustain cancer progression [9]. Unlike conventional small-molecule drugs, peptides can effectively “decode” cancer’s complex molecular signalling network.

### 2.1. GnRH Analogues (Agonists and Antagonists)

Gonadotropin-releasing hormone (GnRH) stimulates pituitary release of luteinising hormone (LH) and follicle-stimulating hormone (FSH), regulating gonadal function and reproduction [76,77]. Its overexpression in various cancers—including breast and prostate—makes it a compelling therapeutic target [78].

GnRH-R1, a GPCR overexpressed in reproductive cancers, enhances tumour-selective drug delivery due to its high affinity for PDCs and inherent antiproliferative effects [18,79,80]. In contrast, GnRH-R2 is largely considered a nonfunctional pseudogene in humans, questioning its role in cancer therapy [81,82]. Further research is needed to clarify its biological relevance.

**Figure 2:**
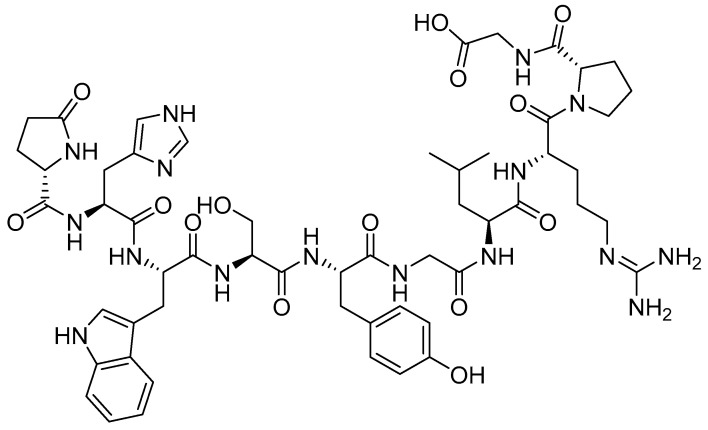
GnRH chemical structure. First amino acid is Glp; pyroglutamic acid (also known as PCA, 5-oxoproline, pidolic acid).

Several GnRH agonists and antagonists have received FDA approval since 1989 (Table 2) [9]. As a key hypothalamic decapeptide, GnRH plays a pivotal role in regulating gonadal steroidogenesis and reproductive function through the hypothalamic–pituitary–gonadal axis [76]. Notably, numerous malignancies—including both reproductive and non-reproductive cancers—demonstrate significant overexpression of the GnRH receptor (Figure 2) [83].

This pathophysiological characteristic has been strategically exploited for targeted therapy, wherein GnRH analogues bind to these overexpressed receptors to modulate downstream hormonal signalling pathways [84,85]. The therapeutic effect is achieved through either agonistic (chronic receptor desensitisation) or antagonistic (immediate receptor blockade) mechanisms of action, depending on the clinical context.

**Figure 3:**
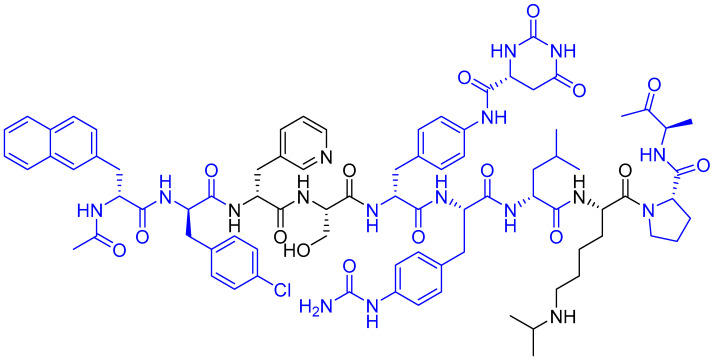
Chemical structure of degarelix (Firmagon). Blue, hydrophobic residues; black, hydrophilic residues.

Degarelix (Firmagon^®^), a second-generation GnRH antagonist, received FDA approval in 2008 for the treatment of advanced prostate cancer [86]. This synthetic decapeptide demonstrates unique physicochemical properties derived from its strategic amino acid composition [86]. The molecule contains hydrophobic residues (including D-ureidoalkyl modifications) that facilitate (i) non-covalent interactions with plasma membranes, (ii) binding to hydrophobic carrier proteins, and protection from proteolytic degradation [87]. Degarelix also has hydrophilic moieties that enable (i) extensive hydrogen bonding networks, (ii) ionic interactions with aqueous environments, and (iii) enhanced water solubility (Figure 3) [87]. This amphipathic design results in superior pharmacokinetic properties, including prolonged circulation time due to reduced renal clearance and remarkable resistance to enzymatic degradation [87]. The molecular architecture of degarelix exemplifies rational peptide drug design, optimising both stability and bioavailability for clinical applications.

Degarelix exerts its therapeutic effect through competitive antagonism of GnRH receptors in the anterior pituitary [86]. This binding immediately suppresses the secretion of LH and FSH, resulting in rapid chemical castration through profound reduction in testosterone production [86]. The consequent androgen deprivation leads to apoptosis of androgen-dependent prostate cancer cells and subsequent tumour regression [86]. Degarelix is administered via monthly subcutaneous injections and demonstrates a favourable pharmacokinetic profile. However, its clinical use is associated with characteristic side effects, including vasomotor symptoms (hot flashes, 56% incidence), injection site reactions (35–40% incidence), and metabolic alterations (weight gain, 5–10% incidence) [86].

While no GnRH peptide analogues are currently in clinical trials for cancer therapy, three non-peptide GnRH receptor antagonists—elagolix, relugolix, and linzagolix—are undergoing clinical trials for endometriosis-associated moderate-to-severe pain. These small-molecule antagonists demonstrate the continued therapeutic potential of targeting the GnRH pathway, though their current applications remain outside oncology [88]. Their clinical success in gynaecologic conditions may inform future cancer drug development, particularly hormone-sensitive malignancies.

### 2.2. Somatostatin (SST) Analogues

**Figure 4:**
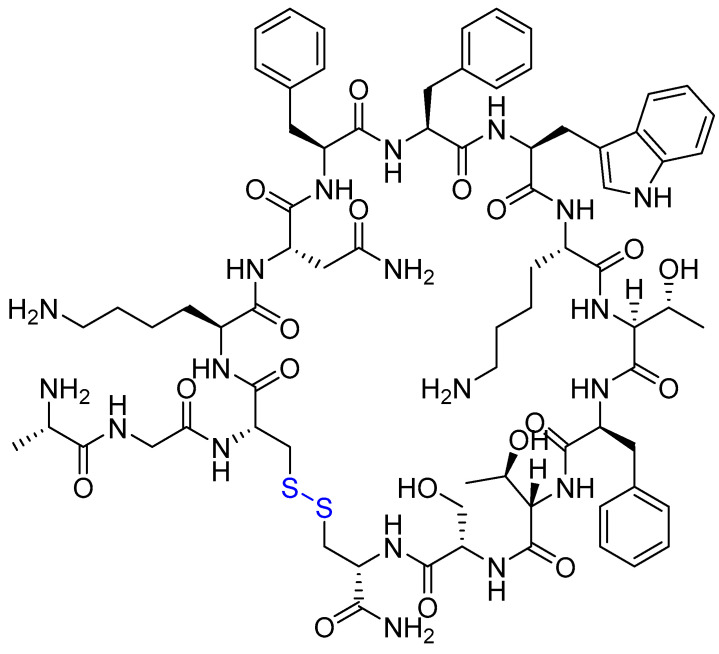
Chemical structure of somatostatin cyclic peptide. Blue, disulfide bridge between Cys3 and Cys14.

SST is a cyclic 14-amino acid neuropeptide that functions as a pleiotropic inhibitory hormone, primarily expressed in the central nervous system (CNS), pancreatic islets, and gastrointestinal mucosa (Figure 4) [89,90].

This multifunctional hormone exerts broad physiological inhibition, including the suppression of hypothalamic–pituitary hormones, the regulation of gastrointestinal secretions (gastrin and gastric acid), and the modulation of pancreatic endocrine function through insulin and glucagon regulation [89,90]. Beyond its endocrine roles, SST demonstrates significant therapeutic potential via three key mechanisms: (i) anti-proliferative effects through cell cycle arrest, (ii) anti-inflammatory properties via cytokine modulation, and (iii) analgesic actions through nociceptive pathway regulation [89]. These diverse biological effects are mediated through five G protein-coupled receptor subtypes (SSTR1–5) that show tissue-specific distribution patterns [89,91]. Notably, SSTR2 has emerged as the predominant mediator of both hormonal suppression and anti-tumorigenic effects, making it a particularly valuable therapeutic target [90]. The clinical relevance of these molecular properties in combating cancer is evidenced by the FDA approval of two synthetic SST analogues between 1998 and 2025 (Table 3), marking an important translation of basic research into therapeutic applications. The structural and functional complexity of SST and its receptor system continues to inform the development of targeted therapies for various endocrine and neoplastic conditions.

**Figure 5:**
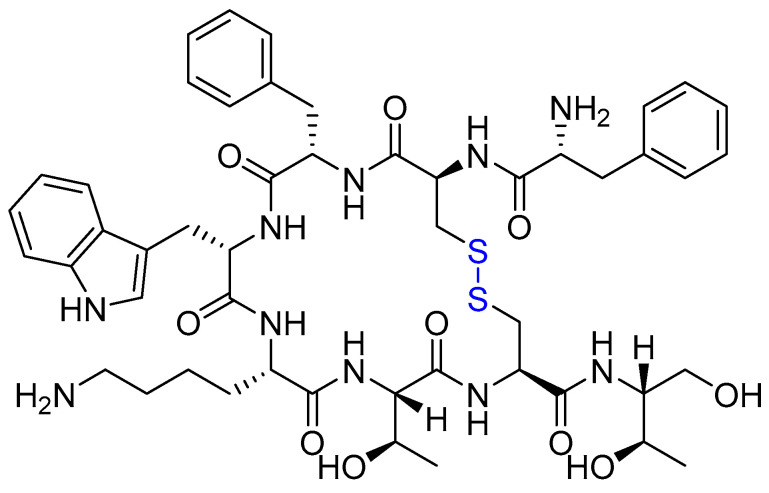
Chemical structure of octreotide. Blue, disulfide bridge.

Octreotide (Sandostatin) is a synthetic cyclic octapeptide mimicking endogenous somatostatin, featuring a Cys2–Cys7 disulfide bridge (Figure 5). Octreotide’s high affinity for SSTRs also makes it valuable for PRRT in neuroendocrine tumours [92]. Octreotide inhibits multiple hormones including growth hormone, insulin, glucagon, and various gastrointestinal peptides [93].

Clinically, it is used to treat acromegaly, thyrotrophinomas, carcinoid syndrome, VIPomas, and severe diarrhoea [93]. Administered subcutaneously, octreotide exhibits prolonged activity compared to natural somatostatin [94]. Common adverse effects include gastrointestinal disturbances (nausea, diarrhoea, steatorrhea), injection site reactions, glucose dysregulation, and cardiovascular effects (bradycardia and arrhythmia) [93]. Cholelithiasis occurs due to reduced gallbladder motility [93].

**Figure 6:**
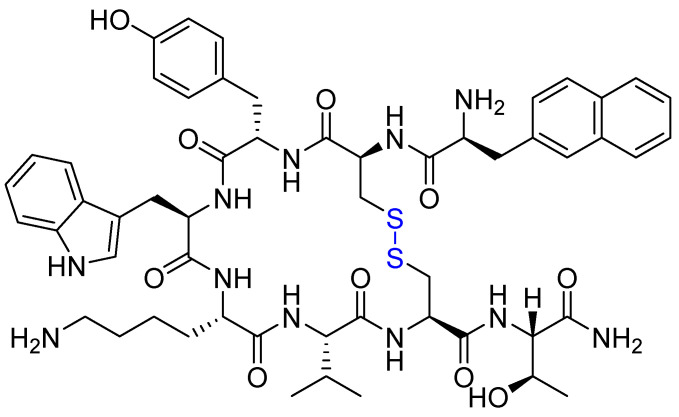
Chemical structure of lanreotide. Blue, disulfide bridge.

Lanreotide (Somatuline^®^) is a synthetic octapeptide analogue of SST, characterised by a stabilising disulfide bridge that enhances its metabolic stability (Figure 6) [9,95]. Approved by the FDA in 2007, it is clinically used for the management of NETs due to its high binding affinity for SSTR2 and SSTR5 [96,97,98].

Upon receptor binding, lanreotide exerts its antitumour effects through multiple mechanisms: (i) inhibition of growth-promoting hormones and peptides, leading to reduced tumour proliferation; (ii) induction of cell cycle arrest; and (iii) suppression of growth hormone (GH) secretion in the pituitary [98,99]. Lanreotide is administered via deep subcutaneous injection, it is associated with a well-defined adverse effect profile, including gastrointestinal disturbances (nausea, 10–20%), musculoskeletal pain (5–15%), cardiovascular effects (hypertension, 5–10%), metabolic alterations (hyperglycaemia, 10–25%), and biliary complications (cholelithiasis, 15–30%) [99].

While not currently applied in oncology, recent advances in SSTR modulation demonstrate its broad therapeutic potential. Paltusotine, the first oral SST2 agonist (in development for acromegaly), showcases how receptor-subtype selectivity and improved pharmacokinetics can enhance treatment efficacy and compliance [100]. Similarly, the SST2-selective agonist CRN02481 effectively controls pathological insulin secretion in hyperinsulinism, proving that targeted SSTR modulation can regulate aberrant endocrine signalling [101]. These successes provide valuable mechanistic insights for oncology applications, particularly for neuroendocrine SSTR overexpression, which is well-documented [97,102]. The demonstrated ability to (1) achieve receptor-subtype specificity, (2) develop non-peptide agonists, and (3) modulate pathogenic secretory pathways offers a strategic blueprint for developing novel cancer therapies targeting SSTR pathways.

### 2.3. Prostate-Specific Membrane Antigen (PSMA) Peptide Antagonist

PSMA is a 100 kDa type II transmembrane glycoprotein, composed of 750 amino acids, which functions as a glutamate carboxypeptidase (GCPII) [103]. It plays a dual physiological role in (i) glutamatergic neurotransmission via hydrolysis of *N*-acetylaspartylglutamate (NAAG) and (ii) intestinal folate absorption through hydrolysis of polyglutamated folates [103]. While PSMA is constitutively expressed in non-malignant tissues—including prostate epithelium, salivary glands, lacrimal glands, and renal proximal tubules—it is markedly overexpressed in prostate cancer cells and the neovasculature of various solid tumours [103]. Moreover, PSMA plays a role in prostate carcinogenesis and disease progression [103]. This overexpression correlates with disease progression, making PSMA a key biomarker and therapeutic target in oncology [103,104].

PSMA-targeting agents exploit this differential expression through selective binding to PSMA-positive cells, enabling both diagnostic imaging and targeted radioligand therapy [105]. Since 2020, the FDA has approved four PSMA-based agents for clinical use (Table 4) [9], reflecting rapid advancements in theranostic applications. Notably, [^18^F]PSMA-1007—a next-generation PSMA inhibitor currently in clinical development—exhibits improved pharmacokinetic properties for PET imaging, including reduced renal excretion and enhanced tumour uptake [23].

**Figure 7:**
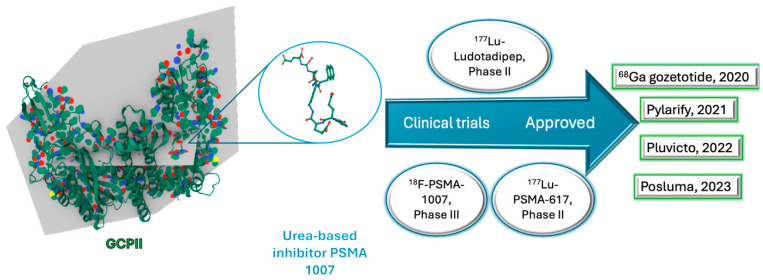
Human glutamate carboxypeptidase II (GCPII) in complex with a urea-based inhibitor PSMA 1007. PDB file; 5O5T [110]. Blue oval, in clinical trials; green rectangle, FDA approved.

All GCPII inhibitors feature three key components: (i) a P1′ moiety binding the S1′ pocket, (ii) a zinc-chelating group replacing the scissile bond, and (iii) an effector moiety interacting with the S1 pocket, with classification based on zinc-binding groups [106,107]. The amphipathic pharmacophore pocket (8 × 8 × 8 Å), formed by residues including Phe209, Asn257, and Tyr700, exhibits limited plasticity due to structural constraints (fixed 8 Å width between Phe209 and Leu428) but contains a flexible glutarate sensor [106,107]. It preferentially binds *C*-terminal glutamate via polar and hydrophobic interactions, explaining why most substrates and clinical inhibitors are glutamate-derived, including those currently undergoing clinical trials (Figure 7) [108,109].

The arene-binding site (Trp541/Arg463/Arg511) enhances inhibitor affinity through π-stacking when accessible [109]. Zinc coordination primarily involves Cys/His/Asp/Glu residues (96% of cases) [109]. The dynamic non-pharmacophore pocket forms a 20 Å-deep entrance funnel (8 Å narrow base) lined by Ser454-Tyr552 and an arginine patch (Arg463/534/536), with Arg463 located on β-strand β13 and Arg534/536 on antiparallel β14 [107].

**Figure 8:**
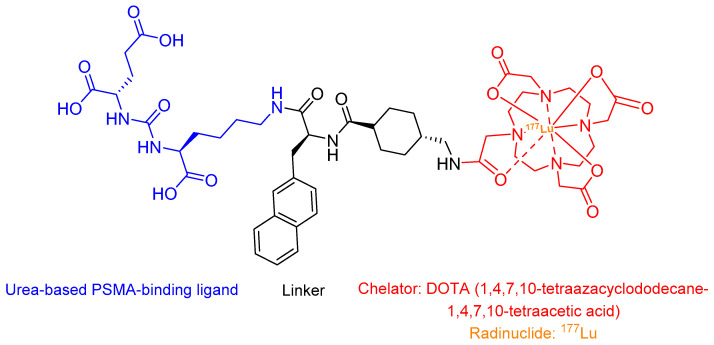
The chemical structure of Pluvicto.

Lutetium (^177^Lu) vipivotide tetraxetan (Pluvicto^®^) is a targeted radioligand therapy comprising three key components: (i) a PSMA-binding urea-based ligand, (ii) a DOTA (1,4,7,10-tetraazacyclododecane-1,4,7,10-tetraacetic acid) chelator, and (iii) the β-emitting radionuclide ^177^Lu (Figure 8) [9,96].

Pluvicto was approved in 2022 to treat metastatic mCRPC with PSMA expression [105]. Pluvicto selectively delivers cytotoxic radiation to PSMA-positive tumour cells and their microenvironment [105]. The emitted β particles induce DNA double-strand breaks in targeted cells and adjacent tumour stroma (bystander effect), resulting in irreversible cellular damage and apoptosis [111]. Pluvicto is administered intravenously and has side effects such as decreased levels of lymphocytes, leukocytes, platelets, calcium, sodium, and haemoglobin [111].

#### ^18^Fluorine-PSMA-1007

**Figure 9:**
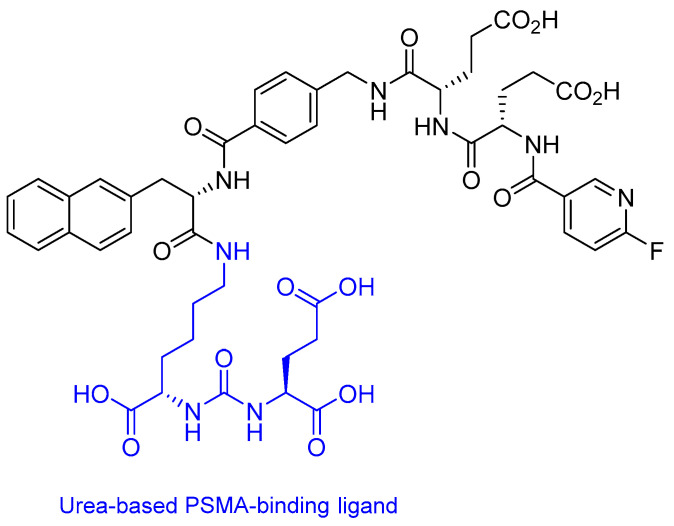
Chemical structure of 18 F-PSMA-1007.

^18^F-PSMA-1007 is an emerging radiopharmaceutical diagnostic agent comprising a PSMA-targeting ligand conjugated to the positron-emitting isotope fluorine-18 (^18^F) (Figure 9) [112]. Currently under clinical investigation, this compound shows particular promise for early-stage prostate cancer detection, demonstrating superior sensitivity for stage I disease compared to conventional imaging techniques [112].

Its diagnostic mechanism relies on selective binding to overexpressed PSMA receptors in prostate cancer cells, enabling precise tumour localisation through ^18^F-mediated PET imaging [23]. While preliminary studies suggest potential theranostic applications, further research is required to fully characterise its therapeutic utility [23]. A notable diagnostic consideration is the compound’s PSMA-specificity, which—while advantageous for tumour targeting—may lead to false-positive interpretations due to physiological PSMA expression in other tissues including bladder transitional cell carcinoma, renal cell carcinoma, and colonic carcinoma [112]. This underscores the importance of correlating imaging findings with clinical and histopathological data to ensure accurate diagnosis. The agent’s high tumour-to-background ratio offers significant advantages in detecting low-volume and metastatic disease, though clinicians must remain cognizant of its limitations in specificity [112].

^18^F-PSMA-1007 exhibits superior pharmacokinetic properties compared to ^68^Ga-PSMA agents, most notably its extended 110-min half-life [23]. This prolonged half-life enhances diagnostic accuracy by allowing for delayed imaging windows, which improves tumour-to-background contrast and increases detection rates [23]. The compound’s favourable physical characteristics—including low positron energy (0.635 MeV) and high positron yield (97%)—provide exceptional spatial resolution, enabling precise identification of small lesions (<5 mm) on PET-CT imaging [23]. Furthermore, ^18^F-PSMA-1007 demonstrates remarkable specificity (85–92%) and selectivity for locoregional lymph node metastases, significantly improving diagnostic confidence in staging and restaging prostate cancer [112]. These combined attributes make ^18^F-PSMA-1007 particularly valuable for detecting oligometastatic disease and guiding treatment decisions [112].

^18^F-PSMA-1007 demonstrates unique pharmacokinetic advantages, including predominant hepatobiliary excretion that minimises urinary tract activity, thereby significantly improving diagnostic accuracy for pelvic lymph node metastases [23,112].

Administered intravenously, this radiotracer achieves optimal imaging quality within 2 h post-injection—a faster timeframe compared to the 3 h requirement for ^68^Ga-PSMA-11 [112]. The rapid target-to-background ratio optimisation enables earlier high-resolution PET imaging, with enhanced detection of small nodal metastases due to reduced obscuration by bladder activity. This improved temporal and spatial resolution makes ^18^F-PSMA-1007 particularly valuable for precise staging of prostate cancer, especially in the prostatic bed and pelvic nodal regions where conventional PSMA PET tracers face limitations due to urinary excretion artefacts [23,112].

Prior to ^18^F-PSMA-1007 administration, comprehensive patient evaluation is required, including an assessment of contrast allergies, vital signs (blood pressure and respiratory rate), and laboratory parameters (biochemical, haematological, and urinalysis) [113]. Renal function is particularly critical, as the radiopharmaceutical is contraindicated in patients with an estimated glomerular filtration rate (eGFR) below 40 mL/min/1.73 m^2^ [113]. Clinical trial data demonstrate an excellent safety profile for ^18^F-PSMA-1007, with no significant adverse effects reported in Phase III studies [113]. Additionally, its cost-effectiveness enhances accessibility for patients [23].

However, diagnostic limitations exist, and false-negative results frequently occur in liver metastases due to high physiological hepatobiliary excretion of the tracer, while false-positive findings may arise from nonspecific bone uptake [112]. These challenges highlight the importance of complementary imaging approaches. Although discordant results were observed in clinical trials, emerging evidence suggests that combined PET/MRI may improve diagnostic accuracy compared to either modality alone [112].

### 2.4. Peptide Receptor Radionuclide Therapy (PRRT)

PRRT represents a precision oncology approach that selectively delivers cytotoxic radiation to tumour cells through the molecular targeting of overexpressed receptors [114]. This therapeutic strategy primarily exploits the high expression of SSTR2 in NETs, utilising either β-emitting radionuclides (e.g., ^177^Lu) or α-emitters (e.g., ^225^Ac) to induce DNA damage in malignant cells while sparing healthy tissue [115]. PRRT demonstrates exceptional clinical tolerability, with a favourable toxicity profile that enhances patient safety during treatment [66,114].

The FDA approved four diagnostic and five theranostic PRRT agents between 1999 and 2025 (Table 5) [67], with several promising candidates currently in development. Notable investigational agents include the following: RAYZ-8009 (DOTA-RYZ-GPC3), targeting glypican-3 in hepatocellular carcinoma [60], and ^177^Lu-FAP-2286, directed against fibroblast activation protein (FAP) in stromal-rich tumours [62]. These therapies show particular efficacy in managing metastatic NETs, where they address the critical need for targeted treatment options in advanced disease [116].

**Figure 10:**
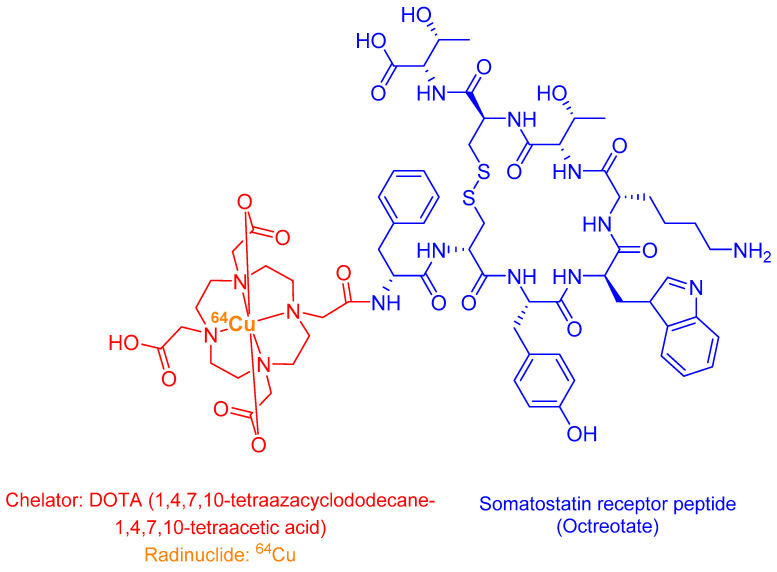
Chemical structure of ^64^Cu DOTATATE (Detectnet).

^64^Cu-DOTATATE (Detectnet™) is a radiolabelled somatostatin analogue comprising three key structural components: (i) a targeting peptide sequence, (ii) the macrocyclic chelator DOTA (1,4,7,10-tetraazacyclododecane-1,4,7,10-tetraacetic acid), and (iii) the positron-emitting copper-64 (^64^Cu) radionuclide (Figure 10) [9,114].

The molecule’s stability is enhanced by a disulfide bridge that constrains its conformational flexibility. Approved by the FDA in 2020 for SSTR PET imaging, Detectnet specifically binds to cells overexpressing SSTR2 with high affinity [117]. The ^64^Cu radionuclide decays via β+ emission (0.653 MeV), enabling high-resolution PET imaging with favourable dosimetry [117].

#### 2.4.1. RAYZ-8009 (DOTA-RYZ-GPC3)

Liver cancer ranks as the sixth most commonly diagnosed malignancy and the third leading cause of cancer-related mortality globally [60,118]. A promising molecular target in hepatocellular carcinoma (HCC) is glypican-3 (GPC3), a cell-surface heparan sulfate proteoglycan that shows minimal expression in normal tissues but significant overexpression in 70–80% of HCC cases [60]. The novel theranostic agent DOTA-RYZ-GPC3 (RAYZ-8009) represents a breakthrough in PRRT, combining a GPC3-targeting macrocyclic peptide with a radiometal chelator for both diagnostic and therapeutic applications [60]. Preclinical studies demonstrate RAYZ-8009’s exceptional cross-species binding affinity for GPC3 across humans, canines, cynomolgus monkeys, and murine models [119].

Comparative preclinical evaluation of ^177^Lu-RAYZ-8009 (β-emitter) and ^225^Ac-RAYZ-8009 (α-emitter) revealed distinct therapeutic advantages: ^225^Ac-RAYZ-8009 delivers high-linear energy transfer (LET) α-particles (5800 keV), exhibits short tissue penetration (40–100 μm), causes clustered DNA double-strand breaks, and demonstrates superior tumour cell kill efficiency per decay [60]. ^177^Lu-RAYZ-8009 shows more pronounced tumour growth inhibition in HepG2 xenograft models, benefits from longer β-particle path length (0.2–2 mm), and enables crossfire effect for heterogeneous tumour targeting [60]. These findings highlight the complementary potential of α- versus β-emitting radiopharmaceuticals in precision oncology, with α-emitters offering superior cytotoxicity for isolated tumour cells and β-emitters providing better volumetric radiation for larger tumour masses.

PeptiDream pharmaceutical company has an ongoing preclinical trial for ^225^Ac/^68^Ga-GPC3 (RYZ 801/RYZ 811). RYZ 811 is a theranostic agent which has the same peptide binder and linker as RYZ 801 but a different radionuclide, ^68^Ga [118]. In total, 47 patients with HCC used RYZ 811, and 90% of them had an uptake of RYZ 811 into the tumour cells [118]. Initial clinical data demonstrated no serious adverse effects in treated patients, supporting the favourable safety profile of RYZ-811 [118]. However, the small cohort size limits the statistical power of these findings, underscoring the need for larger-scale trials to confirm safety and establish more robust efficacy endpoints. Additionally, while preliminary results are promising, further mechanistic studies are required to fully characterise RYZ-811’s diagnostic potential, including its targeting specificity, biodistribution patterns, and detection sensitivity in relevant disease states. These investigations should ideally employ standardised imaging protocols and incorporate comparator agents to validate diagnostic performance.

#### 2.4.2. ^177^Lu-FAP-2286

FAP is a type II transmembrane serine protease exhibiting dual enzymatic activity as both a dipeptidyl peptidase and endopeptidase [120]. This 97 kDa glycoprotein, composed of 760 amino acid residues, belongs to the prolyl oligopeptidase family and demonstrates negligible expression in normal adult tissues [120]. The FAP structure comprises three distinct regions: (i) a short *N*-terminal cytoplasmic domain (6 amino acids), (ii) a hydrophobic transmembrane segment (20 amino acids), and (iii) a substantial extracellular portion (736 amino acids) containing the catalytic machinery [120]. The extracellular domain features two functionally critical subdomains: an 8-bladed β-propeller that regulates substrate access and a *C*-terminal α/β-hydrolase domain housing the catalytic triad (Ser624, Asp702, His734) responsible for proteolytic activity [120].

FAP is selectively overexpressed on cancer-associated fibroblasts (CAFs) in 80–95% of epithelial tumours and certain mesenchymal malignancies, where it plays a multifaceted role in tumour progression [121,122]. FAP-positive CAFs drive tumorigenesis and metastasis through four key mechanisms: (i) promoting extracellular matrix (ECM) remodelling via its collagenolytic activity, (ii) stimulating angiogenesis to support tumour vasculature, (iii) establishing an immunosuppressive TME through cytokine secretion, and (iv) facilitating intracellular signalling pathways that enhance tumour cell survival [123]. This transmembrane protease shows particularly high expression in aggressive cancers including pancreatic ductal adenocarcinoma (>90%), mesothelioma (85–90%), colorectal carcinoma (70–80%), head and neck squamous cell carcinoma (60–75%), and salivary gland tumours (50–60%) [120,124]. The tumour-specific expression pattern of FAP, combined with its central role in maintaining the pro-tumorigenic stroma, makes it an exceptionally promising target for both diagnostic imaging and stromal-targeted therapies, especially in treatment-resistant, stroma-rich cancers where traditional approaches often fail [120].

**Figure 11:**
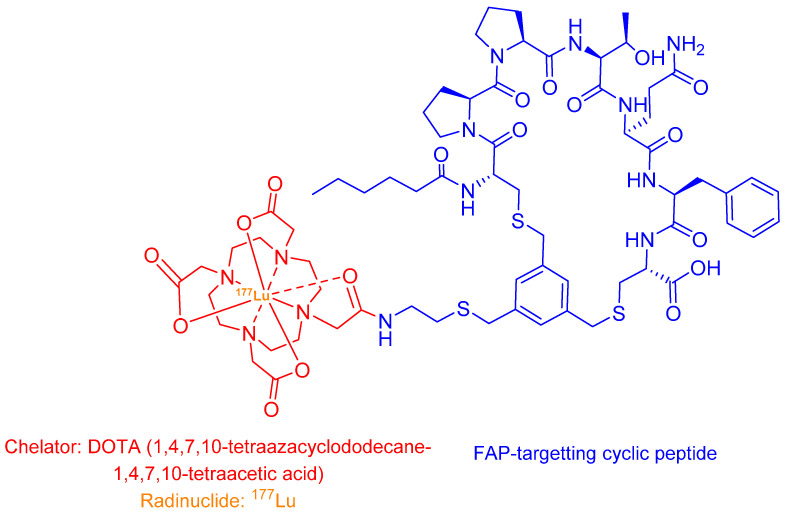
The chemical structure of ^177^Lu-FAP-2286.

^177^Lu-FAP-2286 is a novel theranostic agent comprising a FAP-targeting cyclic peptide conjugated to a radionuclide chelator (Figure 11) [123]. This compound selectively binds to FAP-expressing cells, delivering cytotoxic β-radiation (^177^Lu) for therapy while enabling γ-imaging for treatment monitoring [122,124].

Currently in Phase 1/2 trials, ^177^Lu-FAP-2286 shows particular promise for advanced metastatic sarcomas and other FAP-rich malignancies [16,125]. The diagnostic counterpart, ^68^Ga-FAP-2286, serves a dual purpose: (i) quantifying FAP expression via PET/CT to identify eligible patients, and (ii) providing a baseline for comparative therapeutic response assessment when followed by ^177^Lu-FAP-2286 [123]. This sequential theranostic approach allows for personalised dose optimisation and real-time treatment efficacy evaluation, addressing a critical need in stromal-targeted radionuclide therapy.

^177^Lu-FAP-2286 demonstrates superior tumour retention due to the extended half-life (6.65 days) of lutetium-177, enabling prolonged radiation exposure to FAP-positive malignancies [123]. This pharmacokinetic advantage enhances both diagnostic sensitivity and therapeutic efficacy, as evidenced by robust tumour uptake across diverse cancer types including pancreatic, breast, and sarcomas [123,124]. Clinical data reveal three key benefits compared to its ^68^Ga counterpart: (i) enhanced therapeutic profile, with higher tumour-to-background ratios (3.5:1 vs. 2.1:1 at 24 h), (ii) improved safety, grade 1–2 adverse events only (vs. Grade 3 in 12% with ^68^Ga-FAP-2286), and better clinical efficacy, 62% reduction in metastatic burden (RECIST 1.1), 45% objective tumour size reduction, and 78% pain score improvement [123].

These outcomes correlate with significantly higher patient-reported satisfaction scores (*p* < 0.01), positioning ^177^Lu-FAP-2286 as a transformative theranostic agent for FAP-expressing cancers [123]. The compound’s dual capacity for precise tumour localisation and effective cytoreduction—coupled with its favourable tolerability—underscores its potential to address unmet needs in advanced metastatic disease [123].

^177^Lu-FAP-2286 is administered as a weekly intravenous infusion over six weeks, with continuous monitoring of vital signs (blood pressure, heart rate) and symptom progression throughout treatment [123]. While most clinical studies report only mild (Grade 1–2) adverse events, one trial observed Grade 3 haematologic toxicities—notably leukopenia and pancytopenia—in 9% of participants [123]. The current evidence base remains limited by two critical constraints: (i) an extremely small patient cohort (n = 11, with advanced metastatic sarcoma), which precludes meaningful statistical analysis of efficacy or safety endpoints, and (ii) the absence of a standardised dose-escalation strategy, leaving the maximum tolerated dose (MTD) and optimal therapeutic window undefined [123]. These limitations underscore the necessity for expanded Phase 2 trials incorporating larger, histologically stratified patient populations (n ≥ 50), protocol-defined dose optimisation (e.g., 3 + 3 design), and correlative biomarker studies to establish predictive response parameters. The compound’s promising preliminary safety profile nevertheless supports continued clinical development as a targeted therapeutic for FAP-expressing malignancies [123].

Recent advances in FAP-targeted radiopharmaceuticals include promising preclinical candidates like [^137^La]La-FAP-2286, a novel theranostic analogue currently under investigation [126]. This compound demonstrates the growing interest in FAP as a diagnostic and therapeutic target across various tumour types, building upon the clinical validation of other FAP-directed agents [126]. The theranostic approach exemplified by [^137^La]La-FAP-2286 offers potential advantages for both tumour detection and treatment monitoring in FAP-expressing malignancies, though further characterisation of its binding affinity, pharmacokinetics, and therapeutic efficacy remains necessary [126].

### 2.5. Antibody–Drug Conjugates (ADCs)

ADCs represent a targeted therapeutic platform comprising three key components: (i) a tumour antigen-specific monoclonal antibody, (ii) a chemically stable linker designed to withstand systemic circulation, and (iii) a potent cytotoxic payload (typically 100–1000× more potent than conventional chemotherapy) [1,125,127,128]. These engineered bioconjugates selectively bind overexpressed tumour-associated receptors, enabling precise delivery of their cytotoxic payload directly to malignant cells while minimising off-target effects [1,127]. The FDA has accelerated approval of seven ADCs between 2019 and 2021 for various haematological and solid tumours (Table 6) [9], with several promising candidates like the CEACAM5-targeting SAR408701 currently in late-stage clinical development [31]. This expanding therapeutic class demonstrates how rational drug design can improve the therapeutic index of highly cytotoxic agents through molecular targeting.

**Figure 12:**
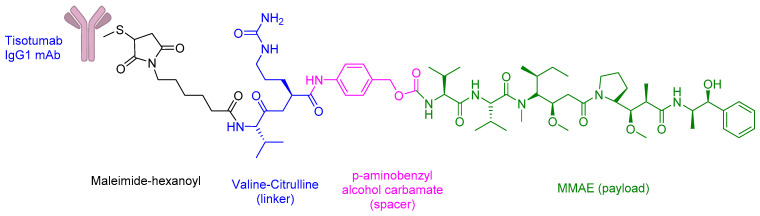
Chemical structure of TIVDAK.

Tisotumab Vedotin-Tftv (TIVDAK) is an FDA-approved ADC with (i) Tisotumab IgG1 monoclonal antibody, (ii) a protease-cleavable valine-citrulline linker, (iii) monomethyl auristatin E (MMAE) payload, which is a potent inhibitor of cell division and microtubule-disrupting agent (Figure 12) [129,130].

TIVDAK has an exceptional antitumour activity and is indicated for recurrent or metastatic cervical cancer [131]. TIVDAK contains a monoclonal antibody that acts against the tissue factor (TF) and a cytotoxic payload, MMAE [130]. The TF is a transmembrane glycoprotein, and it is involved in the initiation of the extrinsic coagulation pathway [130,132]. Additionally, the TF exhibits cell signalling properties, such as the activation of protease-activated receptor 2 (PAR2), which further causes gene transcription, cell survival, and cytoskeletal changes [133]. The TF is expressed on the surface of cancer cells, including cervical cancer, ovarian cancer, NSCLC, breast cancer, head and neck squamous cell carcinoma, and glioblastoma, and is involved in tumour growth, angiogenesis, and metastases [131,134,135].

TIVDAK binds to TF with a high affinity and interferes with the PAR2 pathway [133]. Additionally, TV binds to TF, leading to the formation of a TIVDAK-TF complex [130]. The complex is internalised and transported to the lysosome [130]. The linker is then enzymatically cleaved, and MMAE is released [130]. MMAE binds to the tubulin and disrupts the microtubule polymerisation [130]. This further causes G2/M cell cycle arrest and apoptosis [130]. Additionally, TIVDAK can also provide a bystander effect where its cell permeability property induces the release of MMAE to the neighbouring dividing cells, which in turn causes cell death [129].

TIVDAK is administered intravenously, and its adverse events include nausea (54%), alopecia (39.3%), conjunctivitis (30%), fatigue (26.1%), and dry eye (23%) [130]. Grade 3 or worse treatment-related adverse events include neutropenia, fatigue, ulcerative keratitis, anaemia, and peripheral neuropathy [130]. Additionally, other side effects include haemorrhage, embryo foetal toxicity, and life-threatening Stevens–Johnson Syndrome [130].

**Figure 13:**
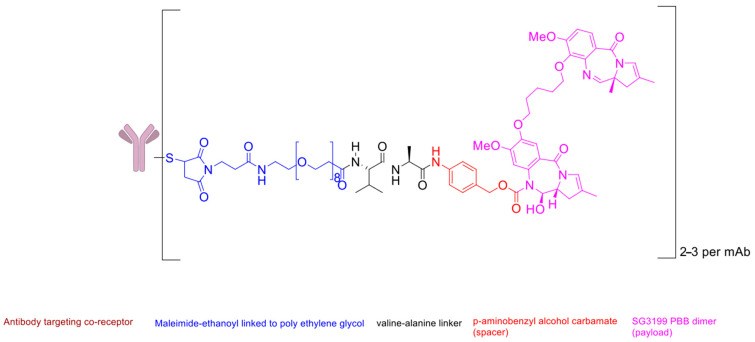
Chemical structure of Zynlota.

Zynlonta^®^ (loncastuximab tesirine-lpyl) is an FDA-approved ADC with three structurally optimised components: (i) a humanised IgG1κ monoclonal antibody targeting CD19, (ii) a protease-cleavable valine-alanine (Val-Ala) dipeptide linker (the sole peptide element in the construct, and (3) a potent pyrrolobenzodiazepine (PBD) dimer payload (SG3199) capable of inducing DNA interstrand crosslinks (Figure 13) [60,136]. Indeed, this drug exemplifies how peptides can serve solely as linkers in ADCs.

This ADC is specifically indicated for relapsed/refractory large B-cell lymphoma (R/R LBCL), including DLBCL not otherwise specified, HGBCL, and DLBCL arising from low-grade lymphoma [136]. The polybrominated biphenyls (PBBs) warhead’s unique mechanism—forming irreversible DNA adducts that block replication—provides exceptional cytotoxicity against malignant B-cells, while the Val-Ala linker ensures stable plasma circulation and tumour-specific payload release via cathepsin cleavage [60].

#### SAR408701

Carcinoembryonic antigen-related cell adhesion molecule 5 (CEACAM5) is a glycosylphosphatidylinositol (GPI)-anchored glycoprotein (180–200 kDa) that plays critical roles in cell adhesion and intracellular signalling [31]. Structurally, it mediates homotypic and heterotypic interactions through its immunoglobulin-like domains, influencing tumour progression and metastasis [31]. CEACAM5 is markedly overexpressed (≥50% of tumour cells) in epithelial-derived carcinomas, with the highest prevalence in gastrointestinal malignancies (70–90% of colorectal, 50–70% of gastric, and 40–60% of pancreatic cancers) [31]. It is also frequently upregulated in NSCLC (30–50%), genitourinary tumours (40–60% of bladder cancers), and a subset of breast carcinomas (15–30%, particularly triple-negative subtypes) [83]. Beyond adhesion, CEACAM5 activates pro-tumorigenic signalling pathways, including PI3K/AKT-mediated cell survival, SRC-family kinase-dependent motility, and Wnt/β-catenin modulation, making it a compelling biomarker and therapeutic target in epithelial cancers [31].

**Figure 14:**
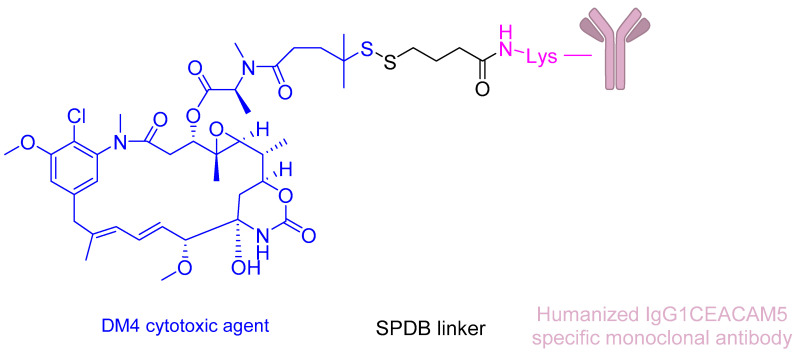
The chemical structure of SAR408701.

In 2023, the investigational antibody–drug conjugate SAR408701 (tusamitab revtamab) advanced to Phase III clinical trials for CEACAM5-positive NSCLC, marking a significant milestone in targeted therapy development (Figure 14) [137].

**Figure 15:**
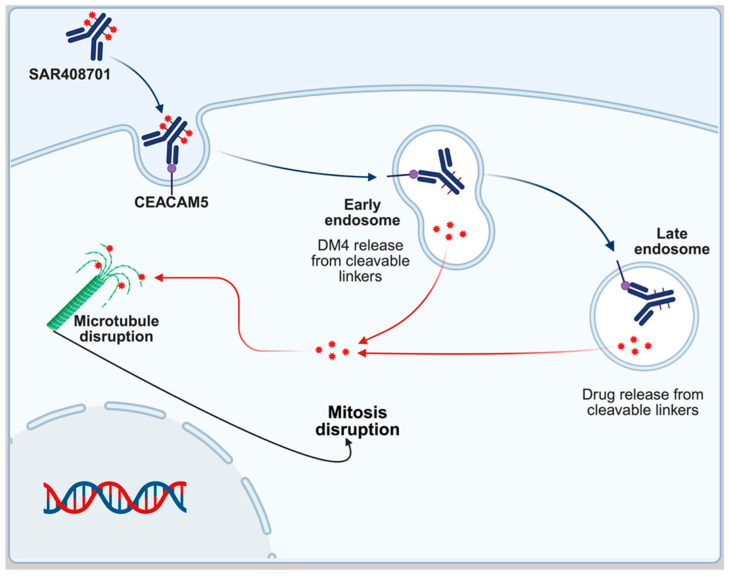
SAR408701 mechanism of action. Humanised IgG1 CEACAM5-specific monoclonal antibody binds to CEACAM5. Once SAR408701 is internalised, the SPDB linker is cleaved, thus releasing DM4. DM4 disassembles the microtubules, thus interrupting mitosis.

This novel anti-CEACAM5 maytansinoid conjugate comprises three key components: (i) a humanised monoclonal antibody specifically targeting CEACAM5-expressing epithelial tumours, (ii) a cleavable sulfo-SPDB linker designed for tumour-selective payload release, and (iii) the potent cytotoxic agent DM4 (a maytansine derivative) [83]. The DM4 payload exerts its antimitotic effect through two complementary mechanisms: (a) binding to tubulin at the vinca domain with picomolar affinity and (b) inducing catastrophic microtubule network disruption (Figure 15) [31].

This dual-action mechanism leads to sustained G2/M phase cell cycle arrest and subsequent apoptosis in CEACAM5-overexpressing malignancies [31]. The ADC’s design leverages CEACAM5’s tumour-restricted expression pattern (minimal in normal tissues) to maximise therapeutic index while minimising off-target toxicity—a critical advantage in treating advanced NSCLC where conventional chemotherapy often shows limited efficacy and significant adverse effects [31].

Preclinical evaluation of SAR408701 demonstrated high binding specificity (KD = 0.5–2 nM) for CEACAM5, with potent dose-dependent antitumour activity in both in vitro and in vivo models, achieving 70–90% tumour growth inhibition in CEACAM5-expressing xenografts through its dual mechanism of antibody-mediated targeting and DM4-induced microtubule disruption [31]. While the ADC showed a strong linear dose–response relationship (*p* < 0.001), therapeutic doses ≥100 mg/m^2^ induced significant toxicities, including haematologic adverse events (Grade 3/4 leukopenia [33%], thrombocytopenia [15%], neutropenia [20%]) and non-haematologic effects (Grade 2/3 asthenia [45%], peripheral neuropathy [18%], and keratopathy [22%]) [137].These findings highlight the critical balance required between SAR408701’s promising antitumour efficacy and its manageable but clinically significant toxicity profile, underscoring the importance of careful dose optimisation in ongoing Phase III trials for CEACAM5-positive malignancies.

### 2.6. Peptide–Drug Conjugates (PDCs)

PDCs represent an emerging class of targeted therapeutics with several advantages over traditional ADCs, including higher selectivity, improved tumour penetration, and reduced systemic toxicity [18,72]. PDCs are broadly categorised into two groups: (1) cell-penetrating peptides (CPPs), which facilitate intracellular delivery, and (2) cell-targeting peptides (CTPs), which bind overexpressed receptors on cancer cells [72]. Structurally, PDCs replace the monoclonal antibody component of ADCs with a smaller peptide (2–20 kDa vs. ~160 kDa for ADCs), enabling deeper tumour stroma penetration and enhanced cellular uptake [138]. Additionally, their low immunogenicity minimises the risk of immune-related adverse effects, a common limitation of ADCs [8,18]. However, PDCs face challenges such as rapid renal clearance due to their short half-life [18,21]. To address this, structural modifications—such as increasing peptide negative charge or incorporating albumin-binding motifs—have been employed to delay glomerular filtration and prolong circulation time [8].

The first FDA-approved PDC, ^177^Lu-DOTATATE (Lutathera^®^), was introduced in 2018 to treat somatostatin receptor-positive gastroenteropancreatic NETs (GEP-NETs) [8]. Administered intravenously, it leverages peptide-mediated targeting for precise radionuclide delivery [139]. Another early PDC, melphalan flufenamide (Pepaxto^®^), was withdrawn from the US market due to insufficient clinical benefit and safety concerns [140]. However, it remains approved in the EU/UK under the brand name Pepaxti^®^ in combination with dexamethasone for relapsed multiple myeloma [141]. Despite these setbacks, multiple PDCs remain in clinical development, demonstrating their potential as next-generation targeted therapies [18].

#### Bicycle Toxin Conjugate (BTC) BT5528

**Figure 16:**
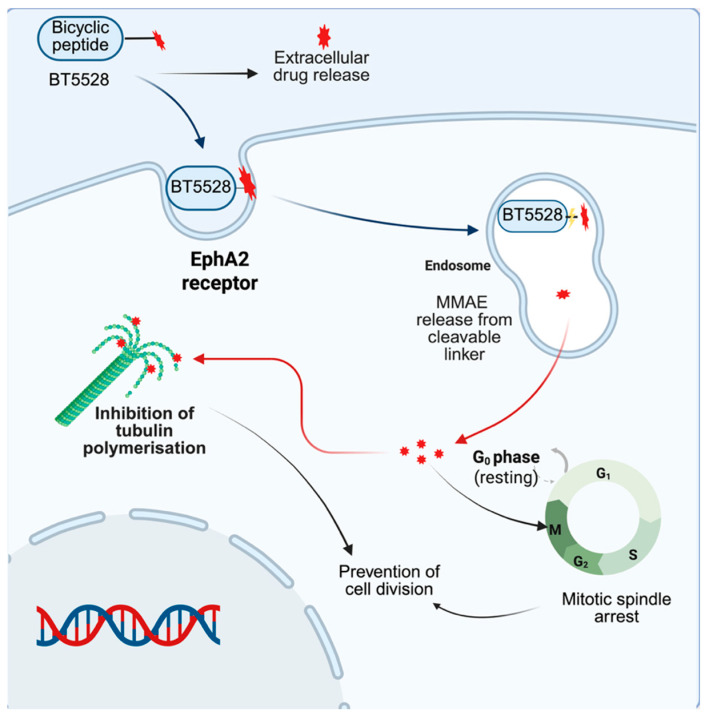
BT5528 mechanism of action. BT5528 targets EphA2, internalises and releases MMAE via protease cleavage, inhibiting tubulin polymerisation and causing mitotic arrest. Extracellular cleavage also releases MMAE into the TME, enabling bystander effects on neighbouring cells.

Solid tumours such as ovarian cancer, glioma, and triple-negative breast cancer exhibit significant overexpression of ephrin type-A receptor 2 (EphA2) receptors compared to healthy tissues [15,33]. Targeting this biomarker, BT5528 represents an innovative BTC composed of three key elements: (1) an EphA2-binding bicycle peptide, (2) a protease-sensitive valine-citrulline linker, and (3) the microtubule-disrupting payload MMAE [13]. Currently in Phase II trials, BT5528’s compact molecular structure (~10 kDa) enables superior tumour penetration compared to bulkier ADCs (~150 kDa), enhancing targeted drug delivery [13]. The conjugate exerts cytotoxicity through two distinct mechanisms: extracellular cleavage by tumour-associated proteases releases free MMAE into the microenvironment (inducing bystander effects), while receptor-mediated internalisation facilitates intracellular payload release, triggering mitotic arrest via tubulin polymerisation inhibition (Figure 16) [13].

This dual-action design capitalises on EphA2’s tumour-selective expression, minimising off-target effects while maximising antineoplastic activity [62]. Preliminary data demonstrate high receptor specificity and potent efficacy, though further clinical evaluation will establish its therapeutic index [62].

BT5528 demonstrates favourable metabolic stability in both preclinical and clinical settings, albeit with a relatively short plasma half-life (~15–20 h) that necessitates biweekly intravenous administration [13]. The recommended Phase II dose (RP2D) of 6.5 mg/m^2^ every two weeks achieves optimal therapeutic exposure, demonstrating significant antitumour activity with measurable tumour regression in EphA2-positive malignancies [118]. While generally well-tolerated at this dose, BT5528 exhibits characteristic dose-limiting toxicities including gastrointestinal effects (Grade 2/3 vomiting [25%], diarrhoea [30%], nausea [35%]), haematologic complications (neutropenia [Grade 3/4: 20%], anaemia [Grade 2: 15%]), and systemic symptoms (fatigue [Grade 2: 40%], alopecia [15%]) [15]. Notably, 18% of patients develop peripheral neuropathy—a class effect of MMAE-based conjugates—which typically manifests as Grade 1/2 sensory disturbances but may require dose modifications at higher grades [15]. These findings underscore the importance of maintaining the RP2D to balance efficacy with manageable toxicity in ongoing clinical development.

### 2.7. Other Classes

#### 2.7.1. Lumisight (Pegulicianine) and Lumicell Direct Visualisation System (DVS)

The FDA recently approved two innovative diagnostic systems for intraoperative tumour margin assessment: Lumisight™ (pegulicianine) and the Lumicell™ Direct Visualisation System (DVS) [16,31] This integrated theranostic platform combines three key components: (1) the fluorescent optical agent pegulicianine (a PDC that selectively accumulates in malignant tissue), (2) a proprietary handheld imaging device with 1 mm spatial resolution, and (3) artificial intelligence-driven detection software that provides real-time, patient-specific tumour probability mapping [7]. Designed for use during breast-conserving surgery, the system enables surgeons to identify residual tumour deposits (<0.1 cm^3^) with 92% sensitivity and 85% specificity in clinical trials, significantly reducing the need for repeat lumpectomies [7]. The FDA approval was based on multicentre trial data demonstrating a 50% reduction in positive margin rates compared to standard palpation-guided resection [31].

**Figure 17:**
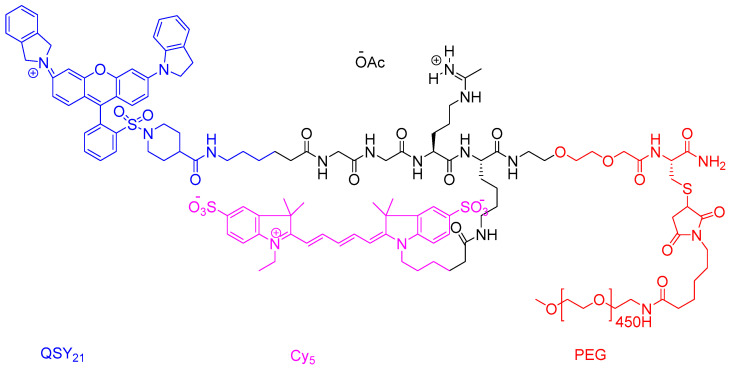
The chemical structure of lumisight. Blue, QSY_21_; pink, Cy_5_; red, PEG.

Lumisight™ is an activatable fluorescent probe with a sophisticated molecular design featuring three key components: (1) a GGRK peptide substrate cleavable by tumour-associated proteases (matrix metalloproteinase (MMP)-2/9 and cathepsins B/L), (2) a near-infrared Cy5 fluorophore (λ_ex_/λ_em_ = 649/670 nm), and (3) a QSY21 quencher molecule linked via polyethylene glycol (PEG) spacers (Figure 17) [16]. In its intact state, the probe remains optically inactive due to fluorescence resonance energy transfer (FRET) between the Cy5-QSY21 pair.

**Figure 18:**
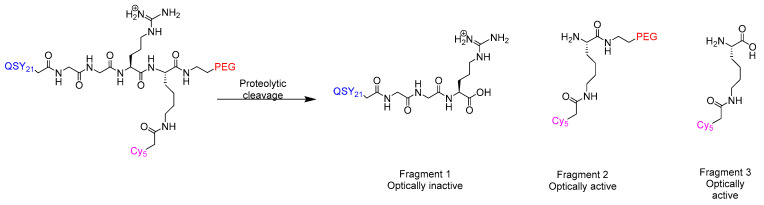
Lumisight cleavage mechanism. Lumisight is a prodrug activated by tumour-associated cathepsins and MMPs, cleaving it into fluorescent fragments (650/675 nm) while the quencher (QSY21) keeps the intact molecule non-fluorescent.

Upon encountering the proteolytic TME, enzymatic cleavage at the GGRK site releases two fluorescent fragments: (i) fragment 2: Cy5-PEG (emitting detectable NIR signal), and (ii) fragment 3: QSY21-PEG-peptide (quenched) (Figure 18) [16].

This tumour-selective activation mechanism achieves >100-fold fluorescence enhancement in malignant versus normal tissue, enabling real-time visualisation of residual tumour deposits with submillimetre precision during oncologic surgery [16]. The design capitalises on the 10–100× higher activity of MMPs and cathepsins in tumours compared to healthy parenchyma, ensuring exceptional target-to-background ratios [16].

Lumisight™ is administered intravenously 2–4 h preoperatively, where tumour-associated proteases activate its fluorescence specifically in malignant tissue [31]. During lumpectomy, surgeons use the Lumicell™ DVS to scan the resection cavity with a handheld probe (1 mm resolution), detecting residual tumour deposits ≥0.1 mm^3^ via real-time AI analysis of the fluorescent signal—a process that reduces positive margin rates by 50% and repeat surgeries by 85%, yielding USD 15,000–USD 20,000 cost savings per patient [31]. While demonstrating high patient satisfaction (92%) and minimal severe adverse effects (<1% Grade ≥3 adverse events), the system requires careful screening for PEG hypersensitivity (0.3% incidence) and contrast allergies (1.2% cross-reactivity risk) [60]. Its primary limitation stems from diagnostic inaccuracy in the pivotal trial (*N* = 357), with 43% false positives (inflammatory tissue) and 8% false negatives (micrometastases), prompting ongoing refinements in imaging algorithms and protease activity thresholds to improve specificity [60]. Despite these challenges, the technology represents a significant advance in precision oncology by enabling immediate, intraoperative margin assessment—a critical factor in reducing local recurrence rates from 5–10% to <2% at 5 years [31].

#### 2.7.2. CLP002 (TR3-M-NP) Peptide in Immunotherapy

Immunotherapy has revolutionised cancer treatment by modulating immune responses, primarily through checkpoint inhibitors targeting pathways like CTLA-4 and PD-1/PD-L1, which normally maintain self-tolerance by regulating T-cell activation [142]. While antibody-based inhibitors (e.g., ipilimumab, nivolumab) have shown remarkable clinical success, their systemic activity often triggers severe immune-related adverse events (irAEs), including pneumonitis, hepatitis, and endocrine dysfunction, due to indiscriminate immune activation [142]. The TME exacerbates immunosuppression by upregulating PD-L1 and MMP-2, which silence CD8+ T-cells and facilitate immune evasion [142].

**Figure 19:**
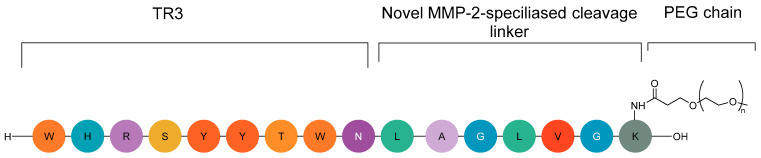
Chemical structure of CLP002.

To address these limitations, peptide-based tumour-activated inhibitors like CLP002 (TR3-M-NP) have been developed—a 9-amino-acid anti-PD-L1 peptide conjugated to a PEG chain that forms protective nanoparticles in circulation (Figure 19) [46,142].

This design leverages MMP-22 overexpression in tumours: upon cleavage, TR3-M binds PD-L1 with high specificity, blocking PD-1/PD-L1 interactions between cancer cells and peripheral blood mononuclear cells (PBMCs) to restore antitumour immunity [142]. Unlike antibodies, CLP002’s low molecular weight enhances tumour penetration while minimising off-target effects in PD-L1-expressing healthy tissues (e.g., heart and lungs), thereby reducing immune-related adverse events (irAE) risks [142]. Despite its promising mechanism, no CLP002-derived agents are currently in clinical development, highlighting an unmet need for smarter immunotherapies that combine precision with reduced toxicity.

## 3. Obstacles and Opportunities

Despite their promise, peptide therapeutics face clinical challenges including poor stability, limited permeability, rapid clearance, and potential resistance [143]. While exhibiting unfavourable pharmacokinetics compared to small molecules [73], peptides offer superior target specificity and reduced off-target effects. Current limitations in membrane permeability and efficacy are being addressed through structural modifications (cyclisation, hydrophobic tuning) and advanced delivery approaches (cell-penetrating peptides, nanocarriers) to accelerate their clinical development [143,144]. Successful examples include cyclic peptides like cyclosporin A (30% oral bioavailability), the cyclic peptide octreotide, which shows improved stability against enzymatic degradation [9] and oral semaglutide (Rybelsus^®^), which maintains efficacy despite requiring 70–140× higher doses than injectable counterparts due to low bioavailability (0.4–1.0%) [145,146].

Tuvia et al. developed an oral suspension (OS) that enhances octreotide absorption by reversibly modulating intestinal tight junctions [147]. Preclinical studies in monkeys confirmed its safety, showing temporary restructuring of tight junction proteins without compromising barrier function [147]. In human trials, oral octreotide/OS achieved comparable pharmacokinetics and growth hormone suppression to subcutaneous injections—a key therapeutic benchmark for acromegaly [148]. This formulation offers a potential non-invasive alternative, potentially improving long-term patient compliance [148].

D-peptides present unique advantages for disrupting cancer-related PPIs, demonstrating high selectivity [149], resistance to proteolytic degradation [150,151,152], and a favourable safety profile [153]. A notable example is the D-peptide NMTP-5, which reactivates p53 by inhibiting MDM2 [154].

## 4. Conclusions

Peptide-based anticancer therapies are revolutionising oncology by merging precision targeting, potent tumour suppression, and reduced toxicity compared to conventional treatments. Innovations such as PRRT (e.g., ^177^Lu-DOTATATE for NETs), PDCs (e.g., BT5528 for solid tumours), and theranostics (e.g., ^225^Ac/^68^Ga-RYZ-801/811 for HCC) enable simultaneous diagnosis and therapy, while diagnostic peptides like Lumisight™ and ^18^F-PSMA-1007 enhance early detection and surgical precision. With five peptide drugs already in Phase III trials (out of 42 reviewed), these therapies demonstrate strong clinical promise—often outperforming traditional benchmarks in efficacy and safety.

However, challenges remain, including rapid clearance (requiring PEGylation or cyclisation), delivery hurdles (necessitating microneedles or nanoparticle formulations), and high costs—highlighted by setbacks like Pepaxto^®^’s withdrawal. Cost-effectiveness is critical, as seen with Lumisight™, which reduces expenses by USD 15,000–USD 20,000 per patient while improving satisfaction. Additionally, mechanistic studies are still needed to optimise agents like RYZ-811, ensuring precise biodistribution and sensitivity in stromal-targeted therapy.

The next-generation peptides are already delivering improved efficacy, safety, and survival, while the success of non-peptide drugs in trials may inspire peptide-based alternatives as preferred modalities. By addressing current limitations through advanced engineering, robust trials, and cost-reduction strategies, peptide therapies could redefine personalised oncology, offering tailored, effective, and patient-centred care with the potential to significantly reduce global cancer mortality.

## Figures and Tables

**Table 1:** Different anticancer peptide therapeutics in the pipeline.

#	Clinical Trials ID	Drug	Indications	Therapeutic Target	Phase	Ref
Diagnostic						
1	NCT06723665	^18^F-PSMA-1007	Prostate cancer	PSMA	III	[23]
2	NCT06520449	^68^Ga-NTA-476	Prostate cancer	PSMA	I	[24]
3	NCT05125016	REGN4336	Metastatic castration-resistant prostate cancer (mCRPC)	PSMA/CD3	I/II	[25]
4	NCT06443762	[^68^Ga] MDM2/MDMX	Positron emission tomography (PET) imaging	MDM2/MDMX	I	[26]
5	NCT03827317	[^18^F]GE-226	HER2 in breast cancer	HER2	N/A	[27]
6	NCT06719856	MMP14 (MT1-MMP) Targeting Bicyclic Peptide Probe	PET imaging in solid tumours	MMP14 (MT1-MMP)	N/A	[28]
7	NCT05518071	FLUOPANC	Fluorescence-guided surgery of pancreatic and bileduct tumours	Integrin	II	[29]
8	NCT02742168	99mTc-3PRGD2	PET in breast cancer	Integrin	N/A	[30]
Therapeutic						
9	NCT02187848	SAR408701	NSCLC	CEACAM5	II	[31]
10	NCT00569257 *	AEZS-108	Endometrial and ovarian cancer	LHRH receptor	Terminated	[32]
11	NCT04180371	BT5528	Solid tumours	EphA2 receptor	I/II	[33]
12	NCT02048059	ANG1005	Brain metastases from breast cancer	LRP1	III	[34]
13	NCT03486730	BT1718	Solid tumours	MT1-MMP protein	I/II	[21]
14	NCT06225596	BT8009	Advanced or metastatic urothelial cancer	Nectin-4	II/III	[35]
15	NCT06730100	CBX-12	Solid tumours, Platinum-resistant and refractory ovarian cancer	Not applicable	II	[36]
16	NCT04740398	CBP-1008	Solid tumour	FRα, TRPV6	I	[37]
17	NCT04928612	CBP-1018	Lung tumour	FOLR1, PSMA	I	[21]
18	NCT02082691	G-202	Hepatocellular carcinoma	PSMA	II	[38]
19	NCT06972628	^177^Lu-PSMA-617	Prostate cancer	PSMA	II	[39]
20	NCT05579184	^177^Lu-Ludotadipep	Prostate cancer	PSMA	II	[21]
21	NCT05465590 **	MB1707	Solid tumour	CXCR4	Withdrawn	[40]
22	NCT01098266	NGR015	Malignant pleural mesothelioma	pAPN	III	[41]
23	NCT02936323	PEN-221	Lung cancer	SSTR2	II	[42]
24	NCT04706962	TH1902	Triple negative breast cancer	SORT1	I	[43,44]
25	NCT06400160	TB511	Advanced solid tumours	M2 macrophages	I/II	[45]
26	NCT05597917	tTF-NGR	Soft tissue sarcoma	CD13, α_v_β_3_ integrin	III	[21]
27	N/A	CLP002	Different cancer types	PD-L1	II	[46]
28	NCT05012618	LUNA18	Solid tumours	GTPases like KRAS	I	[47]
29	NCT03364400	VT1021	Solid tumours	CD36 and CD47	I/II	[48]
30	NCT02264613	Sulanemadlin (ALRN-6924)	Chemoprotection agent	MDM2 and MDM4	N/A	[49,50]
31	NCT06949410	HER2 Vaccine	Locally advanced breast cancer	HER2	I	[51]
32	NCT06789198	Peptide Vaccine	For fibrolamellar hepatocellular carcinoma patients and other tumour entities carrying the driver fusion DNAJB1-PRKACA (FusionVAC22_02)	DNAJB1-PRKACA fusion transcript	I	[52]
33	NCT06762925	METTL3 Peptide Inhibitors	Enhancing anti-tumour immune response by reshaping the TME	CXCL5/CCL5	N/A	[53]
34	NCT06741072	BZLF1 Peptide Vaccine	Prevention of Epstein–Barr virus related cancer in patients awaiting solid organ transplants	The promoters of early EBV lytic genes	Ib	[54]
35	NCT05479045	New York oesophageal squamous cell carcinoma 1 (NY-ESO-1) peptide vaccine	Anti-PD1 resistance in patients with platinum-refractory stage III/IV ovarian cancer (OC)	NY-ESO-1	II	[55]
36	NCT01720836	MUC1 peptide vaccine	NSCLC	Cell surface mucin	I/II	[56]
37	N/A	PQ203 proteinqure	Triple negative breast cancer (TNBC)	sortillin receptor	Ia/b	[57]
38	NCT01967810	ANG1005	High-grade glioma (HGG)	Microtubules	II	[58]
39	NCT03784677	SOR-C13	Advanced solid tumours	Microtubules	I	[59]
Theranostic						
40	NCT06726161	RAYZ-8009/811	Hepatocellular carcinoma	GPC3	I/1b	[60]
41	NCT06991738	177Lu-DOTA-EB-TATE	Adult patients with metastatic, radioactive iodine non-responsive oncocytic (Hurthle cell) thyroid cancer	neuroendocrine tumours (NETs)	I/II	[61]
42	NCT04939610	^177^Lu-FAP-2286	Advanced metastatic cancer	FAP	I/II	[62]

CXCR4, C-X-C chemokine receptor 4; CEACAM5, carcinoembryonic antigen-related cell adhesion molecule 5; EphA2, ephrin type-A receptor 2; FAP, fibroblast activation protein; FOLR1, folate receptor alpha; FRα, folate receptor alpha; GPC3, glypican-3; LHRH, luteinizing hormone-releasing hormone; LRP1, low density lipoprotein receptor-related 1; mCRPC, castration-resistant prostate cancer; MT1-MMP, membrane type 1 matrix metalloprotease; NSCLC, non-small cell lung cancer; pAPN, porcine aminopeptidase N; PD-L1, programmed death-ligand 1; PSMA, prostate specific membrane antigen; SORT1, sortillin; SSTR2, somatostatin receptor 2; TRPV6, transient receptor vanilloid subfamily member 6; tTF, truncated tissue factor. * AEZS-108, The study was terminated because it did not demonstrate significant improvement in progression-free survival (PFS) compared to doxorubicin alone [63]; ** MB1707, the study was withdrawn due to a lack of supporting data for efficacy, safety, and commercial viability.

**Table 2:** GnRH agonists and antagonists that have been approved by the FDA from 1989–2025.

GnRH Analogues	Indication	Approval Year
Agonists		
Goserelin (Zoladex)	Localised prostate cancer	1989
Leuprolide (Lupron)	Advanced prostate cancer and central precocious puberty	1995
Nafarelin (Synarel)	Endometriosis	1998
Trelstar (triptorelin)	Prostate cancer	2000
Histrelin (Supprelin LA)	Advanced prostate cancer and central precocious puberty (CPP) in children	2007
Antagonist		
Ganirelix (Antagon)	Prevent premature LH surges or ovulation in women undergoing fertility treatment of controlled ovarian hyperstimulation	1999
Cetrorelix (cetrolide)	Prevent premature ovulation as part of controlled ovarian stimulation treatment	2000
Abarelix (Plenaxis)	Palliative treatment of advanced prostate cancer	2003
Degarelix (Firmagon)	Advanced prostate cancer	2008

LH, luteinising hormone.

**Table 3:** Somatostatin analogues approved by the FDA from 1998–2025.

Somatostatin Analogues	Indication	Approval Year
Octreotide (Sandostatin)	To treat acromegaly and alleviate symptoms associated with metastatic carcinoid tumours	1998
Lanreotide (Somatuline)	NETs	2007

**Table 4:** PSMA antagonists approved by the FDA from 2020 to 2025.

PSMA Antagonist	Indication	Approval Year
^68^Ga-PSMA-11 (^68^Ga gozetotide)	Diagnosis of prostate cancer	2020
Piflufolastat F-18 (Pylarify)	To detect and monitor prostate cancer	2021
Lutetium ^177^Lu vipivotide tetraxetan (Pluvicto)	To treat adults with a specific type of advanced prostate cancer called PSMA-positive mCRPC	2022
Flotufolastat F-18 (Posluma)	To reveal a more precise image of prostate cancer	2023

**Table 5:** PRRT analogues approved by the FDA from 1999–2025.

PRRT	Indication	Approval Year
Depreotide (Neotect)	To identify SSTR-bearing pulmonary masses in patients presenting with pulmonary lesions on computed tomography and/or chest x-ray who have known malignancy or who are highly suspect for malignancy	1999
^68^Ga-DOTATATE (Netspot)	For diagnosing and staging NETs	2016
^177^Lu-DOTATATE (Lutathera)	To treat NETs	2018
^68^Ga-DOTATOC	Diagnosis and staging of NETs	2019
^64^Cu-DOTATATE (Detectnet)	To aid locating and identifying SSTR-positive NETs in adult patients	2020

SSTRs, somatostatin receptors; NETs, neuroendocrine tumours.

**Table 6:** ADCs approved by the FDA from 2019 to 2025.

ADCs	Indication	Approval Year
Enfortumab Vedotin-Ejfv (Padcev)	Treating adults with urothelial cancer	2019
Polatuzumab vedotin-piiq (Polivy)	To treat adults with diffuse large B-cell lymphoma (DLBCL), a blood cancer affecting white blood cells, in patients whose cancer has not been treated before	2019
Fam-trastuzumab deruxtecan-nxki (Enhertu)	Treating adults with HER2-positive breast cancer that is metastatic (has spread to other parts of the body) or cannot be removed by surgery	2019
Belantamab Mafodotin-Blmf (Blenrep)	Treatment for multiple myeloma, a cancer of the bone marrow	2020
Tisotumab Vedotin-Tftv (TIVDAK)	To treat adults with cervical cancer when the disease has worsened during or after previous systemic (whole-body) treatment	2021
Loncastuximab Tesirine-Lpyl (Zynlonta)	Treatment of adult patients with relapsed or refractory diffuse large B-cell lymphoma (DLBCL) and high-grade B-cell lymphoma (HGBL), after two or more lines of systemic therapy	2021
Telisotuzumab vedotin-tllv (Emrelis)	To treat locally advanced or metastatic, non-squamous non-small cell lung cancer (NSCLC) with high c-Met protein overexpression after prior systemic therapy	2025

DLBCL, diffuse large B-cell lymphoma; HGBCL, high-grade B-cell lymphoma; NSCLC, non-small-cell lung cancer.

## Data Availability

Data sharing is not applicable.
